# Clinical practice of indocyanine green fluorescence imaging in robotic liver surgery – a global expert survey

**DOI:** 10.1007/s00464-025-12553-z

**Published:** 2026-02-09

**Authors:** Noa L. E. Aegerter, Christoph Kuemmerli, Adrian T. Billeter, Caroline Berchtold, Felix Nickel, Cristiano Guidetti, Taiga Wakabayashi, Iswanto Sucandy, Brian K. Goh, Mathieu D’Hondt, Hugo Pinto Marques, Janina Eden, Philipp Dutkowski, Jason Hawksworth, Patrick Starlinger, Beat P. Müller, Philip C. Müller, Robotic Liver Surgery Expert Group, Yuta Abe, Jawad Ahmad, Adnan Alseidi, Koij Asai, Horacio Asbun, Fabrizio Di Benedetto, Ielpo Benedetto, Emrullah Birgin, Ugo Boggi, Ruth Bustamante, Tan To Cheung, Hop Tran Cao, Yee Lee Cheah, Roland Croner, Zoltan Czigany, Nroiyuki Egawa, Alessandro Ferrero, David Fuks, Daisuke Fukumori, Stefan Gilg, Jeroen Hagendoorn, Shinya Hayami, Asmus Heumann, Takao Ide, Taku Iida, Osamu Itano, Jan Philipp Jonas, Yutaro Kato, Felix Krenzien, Philipp Kron, Yoshikuni Kawaguchi, Mickael Lesurtel, Charles Chung-Wei Lin, John B. Martinie, Takuya Minagawa, Zenichi Morise, Yutaka Nakano, Yusuke Nie, Atsushi Oba, Fabrizio Panaro, Florian Primavesi, Nuh Rahbari, Francesca Ratti, Christoph Reissfelder, Ricardo Robles-Campos, Andrea Ruzzenente, Olivier Saint-Marc, Moritz Schmelzle, Daniel Seehofer, Jimin Son, Gregor Stavrou, Stefan Stättner, Benjamin Struecker, Rutger-Jan Swijnenburg, Yu Takahashi, Samer Tohme, Christian Toso, Roberto Ivan Troisi, Stéphanie Truant, Christoph Tschuor, Roeland F. de Wilde

**Affiliations:** 1 Clarunis, University Digestive Health Care Centre Basel, 4002 Basel, Basel, Switzerland; 2 Department of Surgery, Erasmus MC Cancer Institute, Rotterdam, the Netherlands; 3 Klinik Und Poliklinik Für Allgemein-, Viszeral- Und Thoraxchirurgie, Universitätsklinikum Hamburg-Eppendorf, Hamburg, Deutschland; 4 Hepato-Pancreato-Biliary Surgery and Liver Transplantation Unit, University of Modena and Reggio Emilia, Modena, Italy; 5 Department of Surgery, Keio University School of Medicine, Tokyo, Japan; 6 Advent Health Medical Group, Digestive Health Institute at Florida Hospital Tampa/Minimally Invasive and Robotic Surgery, Tampa, FL USA; 7 Department of Hepatopancreatobiliary and Transplant Surgery, Singapore General Hospital and National Cancer Center Singapore, Singapore, Singapore; 8 Surgery Academic Clinical Programme, Duke-National University of Singapore Medical School, Singapore, Singapore; 9 Department of Digestive and Hepatobiliary/Pancreatic Surgery, Groeninge Hospital, Kortrijk, Belgium; 10 Hepato-Biliary-Pancreatic and Transplantation Centre, Curry Cabral Hospital, Centro Hospitalar Universitário de Lisboa Central, Lisbon, Portugal; 11 Department of Surgery, Abdominal Organ Transplantation Division, Columbia University Irving Medical Center, New York, NY USA; 12 Division of Hepatobiliary and Pancreatic Surgery, Department of Surgery, Mayo Clinic, Rochester, MN USA

**Keywords:** Robotic liver surgery, Liver resection, Indocyanine green, Firefly

## Abstract

**Background:**

Indocyanine green (ICG) fluorescence imaging is increasingly incorporated into robotic liver resections (RLR), yet clinical practice regarding timing, dosage, and staining techniques is divergent. This international expert survey aimed to characterize current practices for ICG in RLR.

**Methods:**

Experts in RLR were invited to participate based on surgical volume (experience of ≥ 50 RLR and ≥ 30 annual RLR). A 74-item questionnaire was developed following a literature search and reviewed by a steering committee. The survey addressed indications, timing, dosage, imaging technology, benefits, limitations, training, and future directions of ICG use. Responses collected between September and October 2025 were analyzed.

**Results:**

Seventy experts from 19 countries completed the survey, corresponding to an 88% response rate. Centers performed a median of 180 annual liver resections, including 55 RLR. Most experts used ICG (96%) during RLR. Anatomical demarcation (91%), tumor localization (60%), and biliary anatomy assessment (60%) were the most frequent indications. 60% of experts use preoperative ICG, while intraoperative ICG is mainly administered for demarcation (67%) and biliary tract visualization (40%). Considerable heterogeneity exists in dosage, timing, and staining techniques, particularly in cirrhotic livers and for tumor localization. 53% of the experts had standard operating procedures, whereas 64% expressed the need for a higher degree of standardization. Reported benefits of ICG use included improved anatomical orientation, margin assessment, lesion detection, and support during complex resections. Perceived limitations included background fluorescence, tissue penetration and variable staining in diseased parenchyma. 80% anticipated improved outcomes with combined ICG and three-dimensional image-guidance.

**Conclusion:**

ICG fluorescence is widely used in RLR and is an important cornerstone for precision-guided robotic liver surgery. Standardized clinical practice guidelines, structured training, and technological improvements in imaging and navigation systems are claimed to optimize its clinical use.

Minimally invasive techniques are increasingly adopted in liver surgery, with the robotic approach gaining particular prominence [1–3]. Robotic liver resections (RLR) are considered a safe procedure, offering advantages such as reduced blood loss, conversion rates, and length of hospital stay, compared to both open and laparoscopic approaches [3–5].

For liver surgery, achieving complete tumor removal while preserving sufficient healthy liver parenchyma is essential, particularly in patients with impaired liver function [6]. Additionally, even though surgical outcomes have improved over the years, postoperative complications such as bile leakage can still lead to relevant morbidity [7].

Indocyanine green (ICG) is a fluorescent dye increasingly used to enhance the visibility of hepatic tumors and demarcate the liver parenchyma during liver resections [8]. In liver surgery, ICG can be administered preoperatively for liver function assessment, tumor staining, intraoperatively for demarcation and visualization of anatomical borders through positive or negative staining techniques, visualization of biliary anatomy, or identification of biliary leakage after transection [8]. Inbuilt fluorescence capability enables simple real-time switching to near-infrared mode with robotic systems, thereby facilitating ICG imaging [8].

ICG administration, therefore, has the potential to aid in navigating precision-guided robotic liver surgery, enhancing oncological adequacy while minimizing morbidity [9–11]. Particularly in colorectal liver metastases, ICG has demonstrated significant value by increasing the detection rate of occult lesions and improving the likelihood of achieving margin-negative resections [10, 11]. Despite its increasing use, the administration of ICG in robotic liver surgery remains haphazard, and there is limited evidence on the optimal timing, dose, and administration for different liver parenchymal qualities [12, 13].

The aim of this survey is to assess current clinical practices of ICG use for RLR and to identify potential gaps or areas requiring further standardization.

## Materials and methods

### Participant and institutional characteristics

The survey participants were selected based on their experience in RLR. To be included, experts had to have a previous experience of 50 RLR procedures with 30 RLR annually. Additionally, academic output in minimally invasive liver surgery was required. Eligible experts were invited via email, containing a link to the online survey. As the representativeness of this convenience sample was assumed to be given, as the RLR community is small, this seemed an appropriate sampling technique.

### Survey design

A literature search on the use of ICG in RLR was conducted by two authors (NLEA and PCM). The topics, indications, and timing of ICG use, robotic living liver donation, imaging and technology, benefits and limitations of ICG, outcomes and evidence, standardization and training, and future directions were included in the survey.

All survey questions were newly designed and reviewed, tested and refined in three rounds by the steering committee to ensure clarity, relevance, and completeness. Afterward, the online survey was developed and underwent a second revision by the steering committee. After final revisions, the online survey was sent to the eligible experts. The survey was designed and distributed with SurveyMonkey Inc., San Mateo, California, USA. (www.surveymonkey.com).

This cross-sectional survey consisted of 74 questions and 11 sections, both closed-ended questions (multiple-choice and Likert-type items) and open-ended questions. Questions regarding ICG use in robotic living liver donation were only shown to participants performing such procedures.

Experts were given six weeks to complete the survey, from the 4th of September 2025 to the 26th of October 2025. Two reminders were sent, the first at four weeks after opening and the second two weeks before closure. Double participation or answers after the inclusion period were excluded. Partially completed questionnaires were included in the analysis for available responses.

This study follows the Checklist for Reporting of Survey Studies (CROSS) guidelines[14]. Given the mixed-methods design, reporting was additionally informed by recommendations for mixed-methods research, as applicable[15].

Indocyanine Green Fluorescence Imaging Questions on ICG use included any pre- or intraoperative administration of ICG with the intent to enhance visualization during RLR for benign or malignant liver lesions. This included ICG use for tumor localization, anatomical segmental demarcation, identification of biliary anatomy, detection of bile leakage, and assessment of biliary or parenchymal perfusion. Both positive and negative staining techniques were considered. Centers were also asked whether they adapted ICG protocols based on liver parenchyma quality, disease etiology, or previous neoadjuvant therapy. Questions regarding ICG use for liver function testing were not included, as this survey focused exclusively on the use of ICG for intraoperative guidance.

### Data analysis

#### Quantitative analysis

Data were analyzed using descriptive statistics. Continuous variables were summarized as median and interquartile range (IQR). Categorical variables were reported as counts and percentages and compared using the Fisher’s exact test. All analyses and visualizations were performed with R (R Core Team (2024). R: A language and environment for statistical computing. R Foundation for Statistical Computing, Vienna, Austria. URL https://www.R-project.org/, version 4.4.2).

#### Qualitative analysis

Responses to open-ended questions were analyzed using inductive thematic content analysis. Two authors (NLEA and PCM) independently read all responses to familiarize themselves with the data. A coding framework was developed and applied to the text. Discrepancies were resolved through discussion, with involvement of a third reviewer (CK) when needed. Themes were generated by grouping related topics and were reviewed to ensure they reflected the underlying data. Representative, de-identified quotations were selected to illustrate key themes. Qualitative findings were used to contextualize and explain the quantitative findings, with particular attention to areas of convergence and divergence.

## Results

### Expert panel

A total of 80 RLR experts were invited to participate in the online survey, of whom 70 (n = 88%) completed it. One participant answered the survey anonymously. Respondents represented 58 cities across 19 countries in Asia, Europe, and America. Centers reported a median of 180 (IQR 85–250) liver resections annually, including 55 (IQR 48–100) robotic procedures. Most experts (67/70, 96%) used ICG during RLR, with 49 (70%) applying it routinely (Table 1).

**Table 1:** Indications and timing of ICG application

		Number of respondents (n = 70)	% of all respondents	% of respondents per answer
**Indications and timing of ICG use**				
ICG ever used		67	96	99
Routine use of ICG during RLR		49	70	72
**Indications for ICG use during RLR**				
Tumor localization		42	60	61
Anatomical demarcation		64	91	93
Biliary anatomy determination		42	69	61
Biliary leak identification		20	29	29
Perfusion of biliary reconstruction		7	10	10
Routine use of preoperative ICG administration		42	60	61
**Indications for preoperative ICG (elective RLR)**				
Tumor localization		47	67	68
Anatomical demarcation		10	14	14
Biliary anatomy determination		24	34	35
Liver function test		18	10	10
Routine use of ICG for biliary system visualization		18	26	26
Routine use of ICG after RLR to check for bile leakage		9	13	13
Routine use of ICG after RLR to check liver parenchyma ischemia		15	21	22
**Established SOP for ICG use in RLR**				
Yes		37	53	54
Other		2	3	3
**Preoperative ICG administration during RLR for benign diseases**				
Routinely		15	21	22
Selectively		31	44	45
No		23	33	33
**Preoperative ICG during RLR for primary liver tumors**				
Yes		48	69	71
Other		3	4	4
**Preoperative ICG administration during RLR for liver metastases**				
Yes		45	64	66
Other		5	7	7
Preoperative ICG administration for liver function testing		7	10	
Preoperative ICG administration for biliary tree anatomy		3	4	
Timing preoperative ICG administration, hours		70 (1–720)		
**Challenges of preoperative ICG administration**				
Same-day surgery		22	31	32
Patients scheduling		39	56	57
Costs		7	9	10
**Intraoperative ICG administration during RLR for benign diseases**				
Tumor localization		13	19	19
Anatomical demarcation		47	67	68
Biliary anatomy determination		28	40	41
Bile leak identification		15	21	22
Perfusion of biliary reconstruction		6	9	9
No		7	10	10
**Intraoperative ICG administration during RLR for primary liver tumors**				
Tumor localization		20	29	29
Anatomical demarcation		61	87	88
Biliary anatomy determination		30	43	43
Bile leak identification		19	27	28
Perfusion of biliary reconstruction		5	7	7
No		3	4	4
**Intraoperative ICG administration during RLR for liver metastases**				
Tumor localization		21	30	30
Anatomical demarcation		57	81	83
Biliary anatomy determination		25	36	36
Bile leak identification		14	20	20
Perfusion of biliary reconstruction		3	4	4
No		5	7	7
Repeated administration of intraoperative ICG during RLR		11	16	16
**Challenges of intraoperative ICG administration**				
Learning curve		16	23	23
Costs		4	6	6
Workflow disruption		12	17	17
Protocol variability		10	14	14
None		33	47	48

*ICG* indocyanine green fluorescence imaging, *RLR* robotic liver resection

### Survey

#### Indications of ICG use

**Fig. 1:**
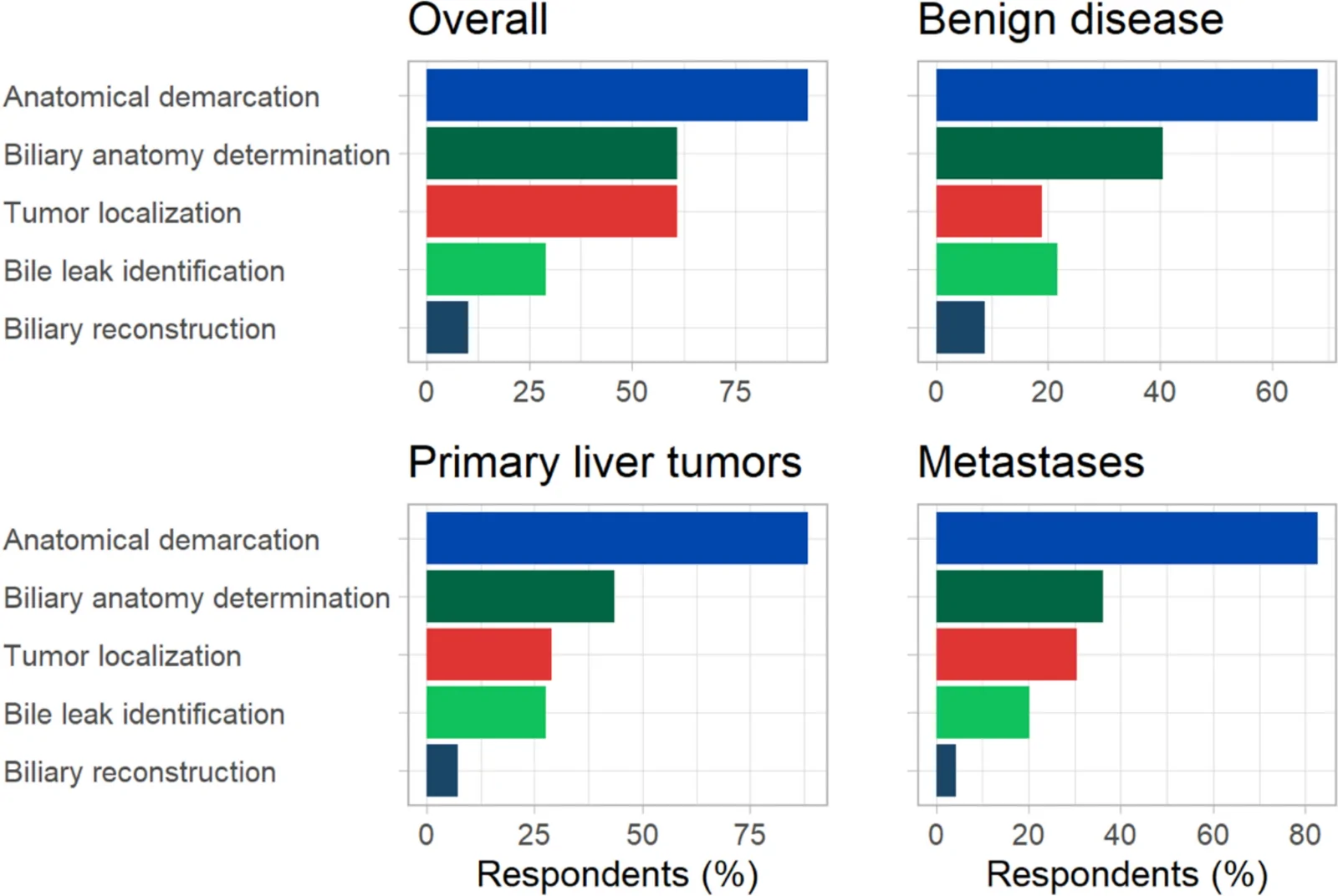
Indications of ICG use for RLR and it illustrates the most common indications for RLR across different liver diseases, with anatomical demarcation and assessment of biliary anatomy being the most frequent applications for ICG overall

The most common indications for ICG use during RLR were anatomical demarcation (n = 64, 91%), followed by tumor localization (n = 42, 60%), and biliary anatomy determination (n = 42, 60%) (Fig. 1). Bile leak detection (n = 20, 29%) and biliary anastomosis perfusion assessment (n = 7, 10%) were less frequent. Routine biliary visualization during RLR was uncommon (n = 18, 26%) and was mainly used for anatomical resections, complex biliary reconstruction, and donor hepatectomy. Only 15 (21%) experts use ICG routinely after completing the resection to assess ischemic liver parenchyma or bile leakage (n = 9, 13%).

##### Standard operating procedures

Standard Operating Procedures (SOPs) for ICG use were available in half of the expert centers (n = 37, 53%). Deviations from SOPs were mainly reported in cases of impaired or ischemic liver parenchyma, iodine allergy, elevated bilirubin levels, or renal dysfunction.

##### Preoperative ICG administration

Preoperative ICG was used by 42 experts (60%), primarily for tumor localization (n = 47, 67%), biliary anatomy assessment (n = 27, 38%), anatomical demarcation (n = 10, 14%), and liver function assessment (n = 7, 10%), (Table 1). As for different liver diseases, preoperative ICG was most commonly used in cases of primary liver tumors (n = 48, 69%), metastatic liver disease (n = 45, 64%) and benign liver disease (n = 46, 65%).

##### Intraoperative ICG administration

Intraoperative ICG was mainly used for anatomical demarcation (n = 47, 67%), biliary anatomy determination (n = 28, 40%), bile leak identification (n = 15, 21%), tumor localization (n = 13, 19%), and biliary anastomosis perfusion assessment (n = 6, 9%). Patterns were similar across disease types (Fig. 1). Less frequent indications included perfusion assessment of biliary anastomosis after resection, hepatic arterial infusion pump perfusion testing, and vascular anatomy delineation.

#### Timing of ICG administration

##### Preoperative ICG administration

**Fig. 2:**
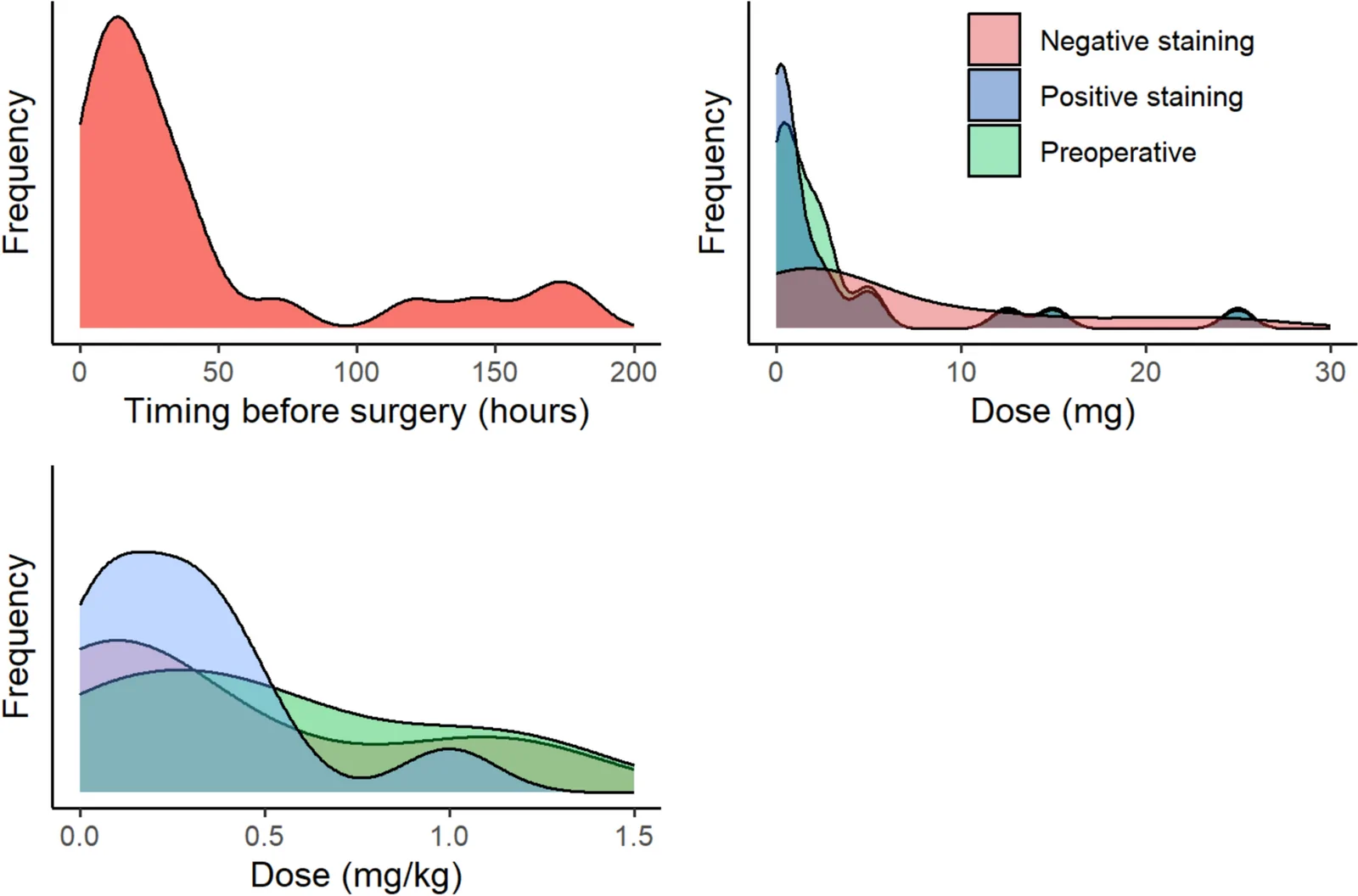
Timing and dosage of ICG Use for preoperative and intraoperative indications. It shows the timing of administration of preoperative staining as well as the administered dosage in mg and mg/kg for the different staining options. Most preoperative ICG administrations occur shortly before surgery, with a strong peak around 10–20 h preoperatively. Across the different types of staining, most experts use a low dosage. Negative staining shows the broadest dosage range

ICG was administered a median of 70 h (1–720) preoperatively, as displayed in Fig. 2. For tumor demarcation, ICG was administered 24 h preoperatively, and 3–4 days in patients with cirrhotic livers. Clinical practice challenges include patient scheduling (n = 39, 56%), same-day surgery (n = 22, 31%), or the costs of the preoperative application (n = 7, 9%).

##### Intraoperative ICG administration

Surgeons administered ICG at the start of the procedure for early anatomical guidance, after Glissonean pedicle clamping or immediately before parenchymal transection for demarcation, on demand during dissection to clarify biliary or vascular structures, or at the end of resection to evaluate perfusion or bile leakage. A few experts reported alternative protocols, including administration 45–60 min before cholangiography or general biliary anatomy assessment. Repeated dosing was used by a minority of experts (n = 11, 16%) to combine positive and negative staining during parenchymal transection and 60 min later or to complement preoperative dosing.

**Fig. 3:**
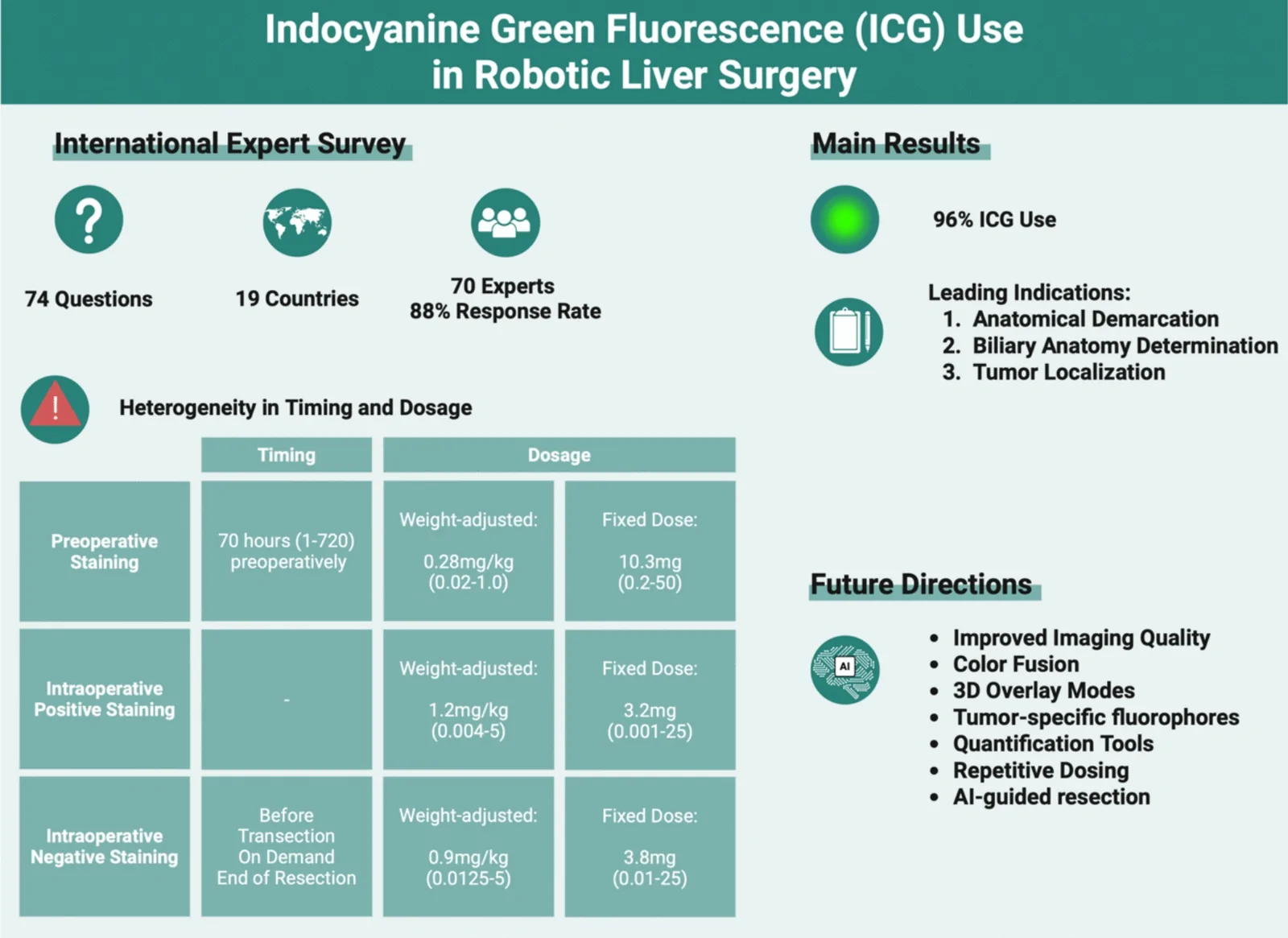
Overview of the ICG use in RLR according to the Robotic Liver Surgery Expert Group. Image created in https://BioRender.com

Intraoperative challenges included the learning curve (n = 16, 23%), workflow disruption (n = 10, 14%), variable protocols (n = 10, 14%), and costs (n = 4, 6%). Importantly, half of the experts (n = 33, 47%) reported no challenges at all (Fig. 3).

### Dosage of ICG administration

#### Preoperative staining

22 experts (31.4%) used weight-based dosing (median 0.28 mg/kg; 0.02–1.0), and 20 (28.6%) used fixed doses (median 10.3 mg (0.2–50), displayed in Fig. 2.

#### Intraoperative positive staining

17 (24.3%) experts used weight-based dosing (median 1.2 mg/kg; 0.004–5), and 24 (34.3%) used fixed doses (median 3.2 mg; 0.001–25). One expert (1%) uses liver volume-based staining (Table 2).

**Table 2:** Dosage of ICG application

	Number of respondents (n = 70)	% of all respondents	% of respondents per answer
Dosage of ICG-administration			
**Preoperative dose**			
Based on body weight	22	31.4	52.4
Fixed dose	20	28.6	47.6
Based on body weight, median (range) in mg/kg	0.28 (0.02–1.0)		
Fixed dose, median (range) in mg	10.3 (0.2–50.0)		
**Intraoperative dosage (firefly system) for positive staining (mg/kg)**			
Based on body weight	17	24.3	41.5
Based on liver volume	1	1	2.4
Fixed dose	24	34.3	58.5
Based on body weight, median (range) in mg/kg	1.2 (0.004–5.0)		
Fixed dose, median (range) in mg	3.2 (0.01–25.0)		
**Intraoperative dosage (firefly system) for negative staining (mg/kg)**			
Based on body weight	23	32.9	42.6
Per body	31	44.3	57.4
Based on body weight, in mg/kg	0.9 (0.0125–5.0)		
Per body, median (range) in mg	3.8 (0.01–25.0)		
Protocol available for cirrhotic liver parenchyma	10	14	16
Protocol available for benign liver disease, primary liver tumors, liver metastases	4	6	6
Protocol available for tumor localization	12	17	19
Protocol available for parenchyma demarcation	15	21	25
Negative staining	44	63	71
Positive staining	7	10	11
Both	11	16	18
Different administration protocol for ICG bile leak identification	6	9	10

*ICG* indocyanine green, *NAC* neoadjuvant chemotherapy

#### Intraoperative negative staining

23 (32.9%) use weight-based dosing (median of 0.9 mg/kg; 0.0125–5) for negative intraoperative staining, and 31 (44.3%) use a fixed dosage (median 3.8 mg; 0.01–25).

#### Dosage for cirrhotic liver parenchyma

Ten experts (14%) used a cirrhosis-specific protocol, generally administering ICG 3–7 days preoperatively (range 2–10 days). Some recommended higher doses or extended time intervals to account for delayed hepatic clearance.

#### Dosage for tumor localization and parenchyma demarcation

Twelve experts (17%) reported using a specific protocol for tumor localization, three of whom administered ICG 24–48 h preoperatively. Some recommend 2–7 days for normal livers, administration at admission, or within 10 days if integrated into functional testing.

15 (21%) experts used a specific protocol for parenchyma demarcation. Most administer ICG after inflow control, typically after Glissonean pedicle clamping. Doses ranged from 0.01 mg/kg to a fixed dose of 2-5 mg. Additional small doses were sometimes given, if demarcation was inadequate. Demarcation was achieved by 44 experts (63%) using negative staining, by 7 (10%) using positive staining, and by 11 (16%) using both methods.

#### Bile leak identification

Six (9%) experts use specific protocols for bile leak detection, ranging from 0.01 mg/kg to 2.5 mg preoperatively.

#### ICG use for robotic living liver donation

Among eight experts (11%) performing robotic donor hepatectomy, six (75%) use ICG before bile duct transection, five (71%) use ICG in place of cholangiography, and four (80%) recommend ICG guidance as the standard approach for bile duct transection in robotic donor hepatectomy.

#### Imaging and technology

Most experts considered current robotic imaging sufficient (n = 38, 54%), as shown in Table 3. Reported limitations included persistent background activity, suboptimal color grading, poor Firefly image quality, and the absence of image overlay or color-fusion. Additional concerns included the lack of quantitative monitoring, limited sensitivity to microdosing, insufficient blood-flow quantification, and distance-dependent or unclear imaging. Additional limitations involved limited depth penetration, challenges with timing, dosing, and interpretation, the single-use nature of ICG, and logistical challenges in preoperative ICG administration. Individual comments focused on inconsistent staining in diseased livers, a fatty hilum, false-positive lesions, residual ICG, mismatch with true tumor burden, and difficulty applying ICG in complex biliary anatomy or after portal vein embolization.

**Table 3:** Future perspectives

	Number of respondents (n = 70)	% of all respondents	% of respondents per answer
*Imaging and technology*			
**Sufficiency of firefly**			
No	13	19	20
Partially	14	20	22
Yes	38	54	58
**Influence of ICG on surgical decision making intraoperatively**			
Rarely	5	7	8
Occasionally	30	43	46
Frequently	16	23	25
Very frequently	14	20	22
Detection of occult lesion due to ICG			
Never	5	7	8
Rarely	25	36	38
Occasionally	26	37	40
Frequently	3	4	5
**Change of resection planes due to ICG administration**			
Never	1	1	2
Rarely	15	21	23
Occasionally	40	57	62
Frequently	5	7	8
Very frequently	4	6	6
**Detection of additional biliary leakage due to ICG administration**			
Never	7	10	11
Rarely	37	53	58
Occasionally	8	11	13
Frequently	2	3	3
Very frequently	1	1	2
**Benefits and limitations**			
Contraindications		41	45
Iodine allergy	29	10	11
Severe renal function impairment	7	4	5
Hyperthyroidism	3	6	6
Other	4	30	33
None	21		
**Outcomes and evidence**			
Improved surgical outcomes or margin status using ICG		39	42
Yes	27	41	45
No	29	11	13
Other	8	39	42
Influence of ICG on learning curve RLR	27	64	69
Necessity of standardized guidelines for ICG use in RLR	45	19	20
Standardized training for RLR team on the use of ICG available	13		
**Future directions**			
Role AI or augmented reality in conjunction with ICG	44	63	70
Reasons that hinder ICG use in hospital		13	13
Costs	9	11	12
Limited tissue penetration	8	16	16
False positive/negative results	11	17	17
No quantitative measurements	12	50	51
None	35	80	90
Improvement of Combination of ICG and image-guidance (3D imaging), 1 = not helpful, 10 = very helpful, median IQR	56, 8 (6.9–9)	1	2
Other	1		

*ICG* indocyanine green fluorescence imaging

### Benefits of ICG use

ICG influenced surgical decision-making occasionally (n = 30, 43%), frequently (n = 16, 23%), or very frequently (n = 20, 23%), according to the participants. In particular, the occasional detection occult lesions was mentioned (n = 51, 73%). A majority of experts altered their resection planes at least occasionally after ICG administration (n = 40, 57%), while additional biliary leakage is detected in some cases (n = 37, 53%).

Reported benefits included clearer anatomy and transection plane, better tumor detection – particularly for small lesions, vanishing metastases, and lesions in cirrhotic livers – margin guidance, and biliary anatomy visualization. One expert also noted improved handling of positive staining in RLR compared with laparoscopic liver resection.

### Contraindications of ICG use

The main contraindications to administering ICG included iodine allergy (n = 29, 41%), severe renal impairment (n = 7, 10%), or hyperthyroidism (n = 3, 4%). 21 (30%) experts reported no contraindications.

### Outcomes and evidence

Improved outcomes or margin status were reported by 27 experts (39%). Several experts mentioned insufficient evidence. 27 experts (39%) mentioned a positive influence of ICG use on the learning curve, enhancing anatomical understanding, facilitating identification of resection planes, and assisting in deciding between anatomical and non-anatomical resection. Experts also highlighted improved confidence during complex cases, better localization of intraparenchymal lesions, and simplified cholangiography.

### Standardization and training of ICG use

45 experts (64%) stated the need to standardize ICG use, including defining indications, dosing, timing, and repeat administration. Experts proposed guidelines in the form of SOPs, Delphi consensus statements, or international guidelines, while some favored flexible, indication-based protocols highlighting that optimal dosing and timing may vary with patient factors, e.g., obesity, fibrosis, cirrhosis, and the intended purpose of ICG.

A minority (n = 13, 19%) reported having standardized team training for the use of ICG in RLR. Training formats include instruction on dosing and timing or SOP-based exercises, participation in structured courses such as the TR300 hepatectomy and Robotic Curriculum for young Surgeons (RoCS) programs[16], and regular hands-on practice, such as performing cholecystectomies, hepatico-jejunostomies, and liver resections in pig models.

### Future directions

Experts prioritized improved imaging quality, color-fusion and overlay modes, tumor-specific fluorophores, multi-color systems, quantification tools, and repetitive dosing capabilities. Forty-four (64%) experts envisioned a role for AI in conjunction with ICG imaging, by improving demarcation accuracy, lesion detection, organ visualization, and operative navigation. Suggested advancements included 3D overlays and fusion with MRCP/CT reconstructions. 35 (50%) of experts see no barriers to ICG adoption in their institutions, 12 (17%) experience limitations due to no quantitative measurements, 11 (16%) due to false positive or negative results, 9 (13%) due to costs, 8 (11%) due to limited tissue penetration and logistics.

56 (80%) think that the combination of ICG administration and 3D image-guidance during RLR would improve outcomes, particularly in complex or parenchyma-sparing resections, donor hepatectomy, and cirrhotic livers. Most experts consider intraoperative image-guidance in RLR very helpful (8, 6.9–9.0).

## Discussion

This international survey of 70 robotic liver surgery experts from 19 countries provides an overview of current clinical practices, benefits, and limitations of ICG fluorescence imaging in RLR. ICG is widely adopted in RLR, with 96% of experts reporting its use, and 70% apply it routinely. Despite this widespread integration, substantial variability exists in timing, dosing, and staining techniques as highlighted by this global survey.

The most consistently highlighted value of ICG in RLR was improved anatomical orientation, as previously reported [17, 18]. Its ability to facilitate tumor localization and visualize biliary structures underlines its role as a compensatory tool for the missing tactile feedback in RLR [19]. Most experts reported that ICG meaningfully influences their intraoperative decision-making at least occasionally, particularly in determining resection planes and, to a lesser extent, in identifying occult or small superficial lesions. Its contribution to post-resection quality control, e.g., bile leak detection or remnant parenchyma perfusion, was reported far less frequently but might increase significantly with adequate quantification tools.

A central finding of this study was the heterogeneous administration of ICG in RLR. Only half of the experts reported having SOPs, and both timing and dosing varied substantially. Preoperative and intraoperative staining doses differed between weight-based administration and fixed doses, and the timing of preoperative administration spanned from 1 h before surgery to more than a week in advance. These inconsistencies were reported as one of the challenges to routine clinical ICG use. Standardized reporting of ICG dose, timing, imaging modality, and surgeon experience would substantially improve comparability across studies.

Although the dosing ranges were similar to those in previous studies, experts noted the need for standardized, indication-specific guidelines [12]. Existing protocols rarely account for underlying liver disease or specific indications. Protocols that adapt the ICG use for underlying cirrhosis or are specific for bile leak detection were used by only a few experts and varied substantially in dose and timing. No expert reported having a tailored protocol for patients who had received neoadjuvant therapy. This is clinically relevant, as the literature report nonspecific fluorescence signals in patients with damaged liver parenchyma and higher false-positive rates in older patients and those with cirrhotic or fibrotic parenchymal impairment[8, 9]. For cirrhotic liver parenchyma, one strategy is to administer ICG for 7–10 days preoperatively to adequately clear it from the damaged liver parenchyma and avoid ICG administration on the day before surgery, particularly in patients who have received chemotherapy [12, 19–21]. A recent meta-analysis further highlights the need for indication-based protocols: preoperative systemic ICG administration, typically 24 h before surgery, appears optimal for tumor localization and margin assessment, whereas intraoperative positive staining is applied for segmental demarcation[21].

ICG is considered particularly useful for visualizing the biliary system around the hilar plate during major resections and donor hepatectomies [19]. Among surgeons performing robotic donor hepatectomy, the majority reported using ICG to guide bile duct transection, with several considering ICG fluorescence their preferred method and even suggesting it as a replacement for traditional intraoperative cholangiography. Recent evidence supports this trend, showing improved identification of hilar structures, fewer biliary complications, and reduced donor and recipient morbidity compared to the standard open approach [22]. Nevertheless, comparative studies are needed before ICG can be considered a definitive substitute for standard cholangiography.

One of the main limitations of ICG mentioned is its limited tissue penetration, as ICG can reliably visualize lesions only up to 10 mm [23]. Therefore, it is useful for detecting superficial lesions but limited in guidance for central lesions, unless those are approached during parenchymal transection. One way to overcome this limitation, though, is the combination of high-quality preoperative imaging (MRI, CT), in combination with intraoperative ultrasound (IOUS) and ICG as an adjunct. IOUS is less sensitive for the detection of superficial lesions in the liver parenchyma but effective in identifying central lesions[24, 25]. Together, IOUS in combination with ICG can provide a more comprehensive intraoperative detection strategy.

Another limitation noted by several respondents was that the evidence for improved outcomes or margin status through ICG administration remains insufficient. While literature on improving resection margins with ICG for primary liver tumors is indeed scarce, multiple studies suggest that ICG may increase R0 resection rates in patients with colorectal liver metastases (CRLM). ICG, especially in combination with IOUS, showed superior R0 resection rates[10, 19, 26]. Additionally, a recent meta-analysis found that ICG was not only associated with higher R0 rates but also the identification of a higher incidence of bile leaks in robotic segmentectomies [21]. A recent RCT has even found that ICG significantly increased the number of identified lesions and improved the 1-RFS, but this was investigated in open and laparoscopic procedures, not robotic[11]. Establishing an international registry would be a critical advance, enabling systematic collection of technical parameters (e.g., dose, timing, imaging settings) alongside clinically relevant endpoints such as long-term oncologic outcomes. Once a sufficient evidence base is established, developing an international consensus on indications, application, and interpretation of ICG would be a highly valuable next step.

Few studies have reported the learning curve for ICG use in RLR[27]. Existing evidence suggests that inexperienced centers may have higher R1 rates and lower accuracy in distinguishing true-positive from false-positive lesions, and that approximately 12 cases are required to achieve consistent fluorescence demarcation[10, 21]. In this survey, 39% of respondents noted that ICG facilitates the general surgical learning curve by enhancing anatomical understanding, particularly in complex resections, yet only 19% reported having formal training programs. Notably, several experts identified the learning curve itself as a factor hindering the implementation of intraoperative ICG use. Structured training in ICG dosing, timing, image interpretation, and limitations should therefore be incorporated into hepatobiliary fellowship curricula and dedicated educational courses. Such training opportunities should be supported and facilitated by surgical societies.

Experts also emphasized the need for technological innovations, particularly AI-assisted guidance, color-fusion imaging, 3D overlay, and tumor-specific fluorophores. Nearly all of the experts believe that combining ICG with 3D intraoperative image guidance would improve outcomes, particularly in complex or parenchyma-sparing resections. Recent evidence supports the use of augmented reality integrated with ICG for laparoscopic right hepatectomy in patients with hepatocellular carcinoma, demonstrating reduced intraoperative bleeding and blood transfusion, as well as improved 1- and 3-year RFS[28]. Another promising outlook might be the incorporation of lymphatic mapping in primary liver cancer, especially cholangiocarcinoma, as already implemented in other fields of gastrointestinal surgery[12].

### Limitations

This study has several important limitations. The findings are based on expert opinions rather than objective data, which may lead to subjectivity and recall bias. The sample size of 70 respondents, although highly specialized experts, remains limited and may not fully reflect the exact global practice. Additionally, survey responses may be influenced by center-specific protocols and access to technology, potentially reducing generalizability to less specialized centers.

## Conclusion

This global expert survey confirms that ICG fluorescence is widely integrated into robotic liver surgery but applied with substantial variability in timing, dosing, and technique. Both quantitative responses and expert narratives emphasized the need for standardized, indication-based protocols. While ICG offers clear value for anatomical guidance and operative precision, current limitations in fluorescence imaging and robotic platforms constrain its reliability. Standardized guidelines, structured training, and advances in imaging and navigation technologies will be critical to optimize the use of ICG in RLR.

## Abbreviations

CRLM: Colorectal Liver Metastases
CROSS: Checklist for Reporting of Survey Studies
ICG: Indocyanine Green Fluorescence Imaging
IOUS: Intraoperative Ultrasound
IQR: Interquartile Range
RLLD: Robotic Living Liver Donation
RLR: Robotic Liver Resection
RoCS: Robotic Curriculum for young Surgeons
SOP: Standard Operating Procedure
